# A behavioral design approach to improving a Chagas disease vector control campaign in Peru

**DOI:** 10.1186/s12889-019-7525-3

**Published:** 2019-09-18

**Authors:** Alison M. Buttenheim, Michael Z. Levy, Ricardo Castillo-Neyra, Molly McGuire, Amparo M. Toledo Vizcarra, Lina M. Mollesaca Riveros, Julio Meza, Katty Borrini-Mayori, Cesar Naquira, Jere Behrman, Valerie A. Paz-Soldan

**Affiliations:** 1 Department of Family and Community Health, University of Pennsylvania School of Nursing, 418 Curie Boulevard, 416 Fagin Hall, Philadelphia, PA 19104 USA; 2 Department of Biostatistics, Epidemiology, and Informatics, Perleman School of Medicine of the University of Pennsylvana, Philadelphia, PA USA; 3 Department of Global Community Health and Behavioral Sciences, Tulane University School of Public Health and Tropical Medicine, New Orleans, LA USA; 4 Chagas Disease Working Group, Arequipa, Peru; 5 Facultad de Salud Pública y Administración, Universidad Peruana Cayetano Heredia, Lima, Peru; 6 Department of Economics, University of Pennsylvania, Philadelphia, PA USA

**Keywords:** Vector control, Behavioral economics, Public health, Intervention design, Chagas disease, Peru

## Abstract

**Background:**

Individual behavior change is a critical ingredient in efforts to improve global health. Central to the focus on behavior has been a growing understanding of how the human brain makes decisions, from motivations and mindsets to unconscious biases and cognitive shortcuts. Recent work in the field of behavioral economics and related fields has contributed to a rich menu of insights and principles that can be engineered into global health programs to increase impact and reach. However, there is little research on the process of designing and testing interventions informed by behavioral insights.

**Methods:**

In a study focused on increasing household participation in a Chagas disease vector control campaign in Arequipa, Peru, we applied Datta and Mullainathan’s “behavioral design” approach to formulate and test specific interventions. In this Technical Advance article we describe the behavioral design approach in detail, including the Define, Diagnosis, Design, and Test phases. We also show how the interventions designed through the behavioral design process were adapted for a pragmatic randomized controlled field trial.

**Results:**

The behavioral design framework provided a systematic methodology for defining the behavior of interest, diagnosing reasons for household reluctance or refusal to participate, designing interventions to address actionable bottlenecks, and then testing those interventions in a rigorous counterfactual context. Behavioral design offered us a broader range of strategies and approaches than are typically used in vector control campaigns.

**Conclusions:**

Careful attention to how behavioral design may affect internal and external validity of evaluations and the scalability of interventions is needed going forward. We recommend behavioral design as a useful complement to other intervention design and evaluation approaches in global health programs.

## Background

Individual behavior change is a critical ingredient in efforts to improve global health. While improving access to high quality health services and strengthening health systems capacity have been important foci of global health and development initiatives, in recent decades the “behavioral revolution” has focused attention on how individual behavior and decision-making drive the effectiveness of most global health programs [1, 2]. Effective, evidence-based interventions for infectious disease prevention—e.g., child vaccination, bed nets, HIV treatment regimens—often fail at the last mile due to individual and household decisions, habits, and behaviors [1, 2]. As countries undergo rapid demographic and nutritional transitions [3, 4], the role of behaviors in driving chronic disease risk is further amplified [5].

Central to the focus on behavior has been a growing understanding of how the human brain makes decisions, from motivations and mindsets to unconscious biases and cognitive shortcuts that we all use to simplify a complex world that demands attention and focus [6, 7]. Recent work in the field of behavioral economics and related fields has contributed a rich menu of insights, principles, empirical regularities and strategies that can be engineered into global health programs to attempt to increase impact and reach [8].

For example, present bias, the tendency to place more weight on costs and benefits realized today and less weight on those realized in the future, makes us prioritize immediate pleasure over actions that are in our long-term interest [9, 10]. Correctly-designed financial incentives can leverage present bias by offering immediate, tangible rewards for current health-related behaviors that may be tedious, unpleasant, or otherwise costly. Loss aversion, the tendency to value losses more highly than gains of the same face value, makes us hold onto assets that we might not be willing to pay for again [11]. An incentive structure that endows participants with up-front resources that are forfeited if behavioral targets are not met leverages loss aversion to increase impact.

In parallel with the behavioral revolution, other important trends are transforming global health research and practice. Interest is growing among the global policy community in the power (and limitations) of randomized controlled trials (RCTs) to evaluate the impact of health and development interventions—the so-called “evaluation revolution” [12]. In the past decade, many RCTs of innovative interventions informed by behavioral insights have been funded and fielded, generating policy-relevant evidence to inform future investments in global health programs. Finally, the “design for development” movement has expanded the global health practitioner’s toolkit with new ways to think about innovation, idea generation, user-defined needs, and user experience [13–15].

Datta & Mullainathan [12] and others [16] have identified a systematic “behavioral design” approach to leveraging the potential of behavioral economics insights into health and development programs. Their framework calls for a four-step process that begins well before the design and evaluation of behaviorally-informed interventions. In theory, a rigorous approach to intervention design should improve the effectiveness of public and private-sector health programs. However, the feasibility of behavioral design as a method for global health research remains unknown. We present here our application of behavioral design to a door-to-door Chagas disease vector control campaign in Arequipa, Peru. We begin with an explanation of the campaign context and the motivation to intervene to improve household participation. Next, we describe the design phases of the study. We report both our original intervention designs as they emerged from the design process, as well as modifications that had to be made to the designs during the Test phase (a pragmatic field trial). Lastly, we discuss lessons learned for future research projects and programs using behavioral design.

### Study context: Chagas disease vector control campaign in Arequipa, Peru

Chagas disease has the highest parasitic disease burden in the Americas [17], with an estimated 8 million infected with *Trypanosoma cruzi*, the etiologic agent of the disease [17, 18]. *T. cruzi* is transmitted via contact with the feces of infected triatomine bugs, which harbor the parasite in their guts. Infections have a 20–30% probability of eventually progressing to cardiac or digestive forms of chronic Chagas disease, which are difficult to treat and often fatal [19].

Since 1991, *Triatoma infestans*, the principal insect vector of *T. cruzi* in southern South America, has been the target of an elimination program known as the Southern Cone Initiative [20]. As a result, three countries (Chile [21], Brazil [22], and Uruguay [23]) have been declared free of *T. cruzi* transmission by *T. infestans*. However, the transmission of *T. cruzi* by *T. infestans* remained common in the city of Arequipa, Peru until recently [24]. In some periurban communities of this city, more than 5% of children were infected with *T. cruzi* prior to control activities [25].

Since 2003 the Peruvian Ministry of Health (MOH) has been engaged in Chagas disease vector control in urban Arequipa (population 650,000). The campaign consists of three phases: a preliminary entomological survey to determine which areas will be covered in the campaign; an “attack” phase in which all households targeted for control measures are sprayed with insecticide (usually Deltamethrin) a total of two times at six-month intervals (referred to as Cycle 1 and Cycle 2); and an ongoing period of active and passive surveillance for vector re-emergence. The attack phase also includes community-wide promotion; the extent and nature of the promotion has varied over time as budgets fluctuated. When a specific locality (neighborhood) is scheduled for spraying, houses are first visited by a health promoter one day prior to spraying to inform them about the campaign and how to prepare their home for the insecticide application (large furniture moved away from walls, food and dishes put away, beddings and clothes put away). Ideally, during this visit the promoter also confirms the household’s willingness to have the home sprayed, and schedules this for the following day at an approximate time (e.g., 8 am, 10 am, 12 pm). Due to frequent no-shows and an unpredictable spray schedule, the promoters overschedule each time slot.

On the day of treatment, the health promoter returns to the house to “open” it: ensuring that a household member is home to open the house to the sprayers and confirming that preparations have been made and that sufficient water supply is available to mix the insecticide solution to the correct concentration. The sprayer arrives and spends 1–2 h spraying the home (depending on the size of the house). Following treatment, household members are asked to ventilate the house for 2–3 h.

As the campaign proceeds, several data elements are collected for all households in campaign areas. During the preliminary entomological survey, data are collected on household infestation and the presence of animals and animal corrals in the house and peridomicile. During the attack phase, infestation data are updated, and promoters and the spray brigades record the ultimate campaign outcome for each household or property scheduled for spray: treated (sprayed), refused, closed (meaning promoters and campaign staff were never able to speak to someone who lives in the house), and uninhabited, vacant, or public/commercial property (typically not treated during the campaign).

Recently, the vector control campaign in Arequipa has suffered from declining rates of participation, threatening efforts to eliminate vector-borne Chagas disease [26] and ultimately the success of the Southern Cone Initiative. Given the low participation rates in the spray campaign in the Mariano Melgar district of Arequipa in 2012–2013, there was mutual interest between the Ministry of Health and our research team in increasing household participation in future efforts in other districts. We adopted a behavioral design framework to guide these efforts. Following formative and pilot work [27, 28] and an initial grant submission (2011–2013), the project was awarded a five-year extramural grant from the Eunice Kennedy Shriver National Institute of Child Health and Human Development in early 2014 (R01HD075869).

## Methods

**Fig. 1:**
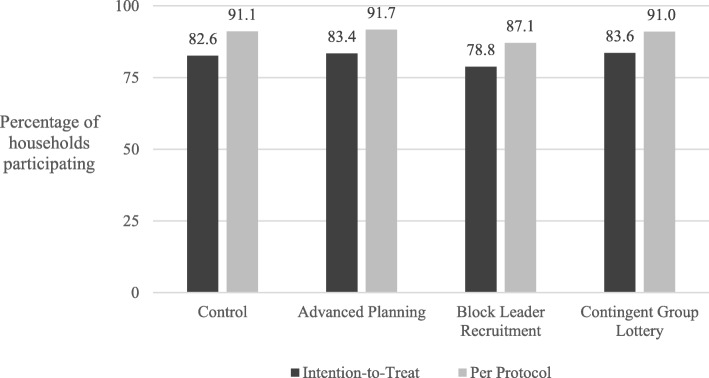
Percentage of households participating in insecticide treatment during Cycle 2 of attack-phase Chagas-disease vector-control campaign, Arequipa, Peru, 2015, by treatment arm and analytic sample. See Buttenheim et al. 2018 for more details

Datta & Mullainathan’s [12] behavioral design approach comprises a four-step process that begins well before the design and evaluation of behaviorally-informed interventions (see Fig. 1 in Datta & Mullainathan). Similar to a product design or engineering process, behavioral design starts with problem definition: what are you trying to fix, and why? Next, a diagnosis phase identifies “actionable bottlenecks” or possible targets of intervention where psychological factors are driving behavior. Only then do the investigators proceed to intervention design, matching behavioral insights to actionable bottlenecks. In the final step, these interventions are tested, ideally in a randomized controlled counterfactual evaluation complemented by a factual evaluation of mechanisms, implementation, and participant experience [29].

### Problem definition

Problem definition drives problem solution [30]. This relationship has important and often overlooked implications for intervention design. Problem definition can be specific to the local context. Problems that are amendable to the behavioral design process are typically related to end-user behaviors (vs. upstream structural barriers), assume a pre-determined solution, and have the potential to significantly improve well-being [31]. The problem definition step is complex for Chagas disease. Definitions of success for the control of Chagas disease have shifted over the years and in some ways the existing vector control campaign functions as a pre-determined solution. We generated and evaluated several possible problem definition statements, and iteratively reviewed with stakeholders and investigators until consensus was reached. The agreed-upon problem definition is reported below in Results.

### Behavioral diagnosis

Various methods were applied to diagnose the problem. As part of the larger parent study, we analyzed participation and survey data collected by the Ministry of Health in the first round of spraying in one district (*n* = 2911 households) [28]. We conducted focus group discussions (2 groups with total of 17 participants) and semi-structured interviews (*n* = 71) with household members in campaign areas in March and May 2013 respectively, in a district where a spray campaign had taken place 1 year earlier [27]. We also relied on knowledge gained in more than a decade of working with MOH on the vector-control campaign. With these multiple rich sources of data, we distilled specific insights about psychological factors that were shaping bottlenecks to our target behavior (see Results section below).

### Intervention design

Following the diagnosis phase, we engaged in an iterative process of grouping actionable bottlenecks together into coherent bundles, and then looking to the existing literature on the application of specific behavioral economic principles to generate intervention designs that could address those bottlenecks. Design work was done collaboratively with stakeholders from many disciplines (e.g., economics, epidemiology, ecology, behavioral science) and perspectives (e.g., researchers, health ministry managers, campaign fieldstaff). As intervention ideas were formulated, some rapid-cycle pilot testing to assess feasibility, acceptability, and effectiveness was done.

### Intervention trial

A cluster-randomized controlled trial was designed and carried out in the Alto Selva Alegre district of Arequipa during the Cycle 2 treatment in March–October 2015 [32]. We divided the spray area into 56 clusters of approximately 80–100 households. Clusters were randomly assigned to a control arm or to one of the three intervention arms, with assignment balanced by prior vector infestation and household participation in a preliminary entomological survey. The sample size in each arm was approximately 1400 households. All households in the sampled sectors were included in the trial.

The primary outcome in the cluster-randomized trial was participation rate by intervention arm in an intent-to-treat (ITT) analysis. The study was powered to compare the relative effectiveness of each intervention to the control (current campaign) and to each of the other interventions. We also planned analyses of secondary outcomes including treatment intensity (number of visits from promoters and sprayers needed to achieve participation) and a per protocol analysis of participation by intervention arm (including only those households which received the intervention).

## Results

### Problem definition

Multiple candidate problem definition statements emerged including the elimination of the insect vector from the study area, or seroprevalence of *T. cruzi* infection in children 5 or younger of below 1%, the keystone of the current WHO/PAHO definition of disruption of vectorial transmission [33]. We also considered the proportion of houses sprayed with insecticide in the spray campaign, a number with important epidemiologic and programmatic significance. However, the problem that seemed most compelling and salient, both for our research and for the success of the campaign going forward, was at an individual level: household members were choosing not to participate in the campaign.

### Behavioral diagnosis

Analysis of prior campaign data revealed geographic clustering of participants along blocks. Specifically, the odds of participating in spraying doubled if one had a neighbor who participated, controlling for socioeconomic status and prior infestation (both of which are spatially correlated) [28]. Although the spatial clustering we observed does not imply that one’s neighbors influence the decision to participate or not in a spray campaign, it does suggest that there may be an element of social contagion [28]. Spray campaign refusers were also asked for a motive for their refusal. The most common reason was that they could not wait for the sprayers because of work obligations, followed by concerns about side-effects of the insecticide (i.e., allergies, asthma triggers) [28].

Focus groups and interviews revealed multiple barriers to participation: inconvenient spray times due to work obligations; the difficulty of preparing homes for spraying (e.g., moving heavy furniture); lack of trust in sprayers (e.g., concern about theft or about sprayers assessing property value to increase taxes); secondary health impacts (e.g., allergic reactions to insecticides); aversion to the odor of the insecticide; lack of ventilation within the home; low perceived Chagas disease risk and perceived need to spray; stained walls; and the inconvenience of waiting outside during insecticide application [27]. As suggested in our prior analysis [28], we also found that most household decision-makers consulted other family members about their decisions to spray the home, and slightly more than half talked to a neighbor about it.

We gleaned additional insights through analysis of several important structural, political, and financing factors that have influenced the design and implementation of the local campaign since its initiation. In the early years, the campaign received technical support from the Pan American Health Organization (PAHO) and financial support from the Canadian International Development Agency (CIDA) [34]. This support allowed for significant investments in campaign promotion via radio ads and murals. In 2002, Peru’s Ley Orgánica de los Gobiernos Regionales transferred numerous functions, including many related to health, to regional governments [35]. The transfer was completed in 2006. Around this time the funding for the campaign was diverted from one regional office (the regional office of the Ministry of Health) to another (the Red de Salud Arequipa-Caylloma). A gap in funding in 2009 prevented the purchase of insecticide and paused the campaign [36, 37]. The bulk of the campaign promotion is now carried out through megaphoning and household visits. Over time, the geographic focus of the campaign has shifted from primarily poor, recently established communities *(pueblos jovenes*) to districts with greater economic and social heterogeneity. Vector infestation patterns are also more heterogeneous in the more urban districts [38–40]. Participation in poorer neighborhoods is generally higher than in wealthier and more established areas of the city.

The results of the behavioral diagnosis phase were summarized in a set of actionable bottlenecks or barriers to our desired outcome (household participation in the vector control campaign). These bottlenecks were linked to specific behavioral economics constructs (see Table 1), and eventually to intervention designs.

**Table 1:** Mapping of bottlenecks to behavioral-economics principles and interventions

	Advanced planning	Block leader recruitment	Contingent group lotteries
Actionable bottlenecks			
Time/schedule constraints	✓		
Logistics (Furniture moving, renters)	✓		
Stigma		✓	✓
Lack of awareness		✓	✓
Stranger in home		✓	✓
Insecticide concerns			✓
Behavioral-economic principles			
Consistency and commitment	✓		
Present bias	✓		✓
Bandwagoning		✓	✓
Framing		✓	
Reciprocity		✓	
Base-rate bias			✓
Regret aversion			✓
Attribution bias			✓

### Intervention designs

After several design iterations, three interventions emerged: 1) advanced planning; 2) block leader recruitment; and 3) contingent group lotteries (see Table 1). The design process unfolded over the course of submitting and resubmitting an extramural grant proposal to fund the study. During the design process we frequently consulted with our field staff, partners at the Ministry of Health (MOH) in Arequipa, and behavioral economics experts. Brief descriptions of each intervention follow. Tables 2, 3 and 4 provide considerably more detail on rationale, bottlenecks addressed, behavioral economic principles applied, and evolution of each intervention from original design to implemented version.

**Table 2:** Advanced planning intervention: Diagnosis and Design

DIAGNOSIS		DESIGN		
Actionable bottlenecks	Relevant behavioral economic principle	Original intervention design element	Implementation challenge	Revised design
Preparing home for spraying is perceived as difficult and stressful.	Present bias will lead households to discount the burden of participating in the future Self-consistency bias [38] suggests that once a household commits in advance, they will be more likely to follow up on that commitment	Schedule households for spraying 2–4 weeks in advance.	2–4 weeks was too far in advance for both households and the spray brigade. Brigade chiefs could not plan that far ahead of time due to water shortages, health sector strikes, holidays, and a canine rabies outbreak. Households scheduled in advance were often not home for spray appointment.	Advance scheduling was revised to 7–10 days ahead of spraying. Spray brigade schedule was intentionally “overbooked” to account for no-shows.
Households are not able to plan for spraying when scheduled only 1 day prior.	Planning prompts can help follow through on desired behavior. [39–41]	Offer planning prompts as well as email, text message, phone call or visit reminders to advance scheduled households.	Few households chose email or text message reminders	Only call or visit reminders were offered.
Those working during the day cannot participate.		Provide more flexible scheduling options to households (evenings and weekends, more choice of spraying time).	Evening hours were not feasible for the spray brigade, although frequently requested by households. Weekend spraying was used for “recuperation,” or to catch up, but could not be scheduled in advance.	Households scheduling in advance could choose preferred appointment times during the regular spray day but not weekend or evening hours.
Sprayer arrival time is unpredictable.		Spray households according to pre-arranged schedule (rather than proceeding house-by-house down a block).	If households scheduled in advance were not at home for spray appointment, sprayers could not “make up” the missed appointments by spraying nearby households, as these were also scheduled in advance.	We staffed up extra sprayers to fill in when regular spray brigades could not accommodate the pre-arranged schedule.

**Table 3:** Block leader recruitment intervention: Diagnosis and Design

DIAGNOSIS		DESIGN		
Identified Actionable bottlenecks	Behavioral economic rationale	Original intervention design elements	Implementation challenge	Revised design
Community members lack of knowledge of campaign.	Campaign t-shirts increase the salience [42] of the campaign and gain-framed messages [43, 44] highlight the benefits of participation.	Two to 4 weeks prior to targeted spray dates, campaign staff approach local leaders and ask them to serve as neighbor recruiters. Provide training to leaders on Chagas disease, the vector control campaigns, and how to promote the campaign to neighbors Recruiters asked to promote the campaign to 10–12 houses on their block through multiple visits, wear the t-shirts regularly, and distribute additional t-shirts.	Recruitment of leaders was very difficult in some neighborhoods, particularly those with less social cohesion. Group training was difficult to schedule. Considerable variation in leader skills and background.	Snowball recruitment methods for leaders were added. Research staff also recruited based on prior personal connections. Group training was shortened; a second one-on-one practice session was added. Leaders were given a training certificate and a recognition ceremony was held at the end of the campaign.
Community members report not knowing others who participate.	Bandwagoning research suggests households will more likely to participate if they are told their neighbors are participating as well. [45, 46]	Recruiters are encouraged to tell households that they participated themselves, and also that neighbors are participation.		
Distrust of the government campaign and campaign field staff	Research on norms and peer pressure suggests that households will be more likely to participate if they are recruited by a neighborhood opinion leader. [47, 48]	Recruiters receive promotional t-shirts with gain-framed messages, a clipboard, a phone card with mobile minutes, and the educational materials used by campaign staff.

**Table 4:** Contingent group lotteries intervention: Diagnosis and Design

DIAGNOSIS PHASE		DESIGN PHASE		
Identified Actionable bottlenecks	Behavioral economic rationale	Original intervention design elements	Implementation challenge	Revised design
Costs (time, lost wages, dislike of insecticide, distrust) are immediate and salient.	Lottery leverages present bias by making the intangible benefits of spraying more immediate [49, 50].	Contiguous households are assigned to lottery groups of 6. Lottery groups are randomly assigned a lottery number. In a lottery drawing following the attack phase of the campaign, households whose lottery group is selected and who participated in spraying will win a small prize such as a food basket.	The Ministry of Health preferred that lottery prizes be related to vector control. Agreed-upon prize (gift card to home repair store) could not be carried out when home store discontinued use of gift cards. Block structure did not always allow for lottery groups of size 6. Houses assigned to lottery groups using campaign maps were occasionally abandoned or had split into multiple households	A gift card to a large home repair store was selected as the lottery prize, to focus winners on purchasing supplies to improve home against vector infestation. A local hardware store agreed to accept vouchers developed by the research team. The hardware store recorded the content of purchases made with vouchers Lottery groups could range in size from 5 to 7 households, allowing for a group to lose or gain a household in the field.
Benefits are intangible, probabilistic, and far in the future.	Immediate feedback and rewards can increase desired behavior.	Lottery prizes are awarded to households as soon as possible after the participation decision.	Unpredictable timing of spray campaign made immediate notification of lottery prizes impossible.	Lottery groups were assigned a lottery date several weeks in the future. Study team visited winning households within a few weeks of lottery drawing.
No perceived cost to refusing participation.	Invoke anticipated regret, leveraging regret aversion [51, 52]	Lottery procedure notifies winning households and non-participating households who could have won.	Households and some field staff assumed the households only entered the lottery if they sprayed.	Ongoing training and fidelity checking of field staff needed.
Spraying perceived as signifying infestation	The lottery may mitigate any stigma associated with participation [53, 54]	Households can attribute spraying participation to lottery incentives.		
Some neighborhoods report little awareness of neighbors’ participation decisions.	Making neighbor participation more salient through creation of lottery groups will increase each household’s motivation to participate. [55]	If all households in the selected group participated, each participating household also wins an inexpensive tablet computer.	The Ministry of Health preferred that lottery prizes be related to vector control.	Lottery prize if all households in the group participated was a voucher twice the value (USD 34) of the individual prize.

#### Advanced planning (see Table 2)

To address challenges in both scheduling treatment at a convenient time and getting the home ready for treatment, this intervention leveraged present bias and planning prompts to encourage households to schedule the spray well in advance, and to make a plan based on this commitment. In coordination with the existing MOH campaign, households were approached 7–10 days in advance and asked to commit to participate. Households that agreed to participate were offered convenient 2h appointment windows on their preferred future spray date. A refrigerator magnet with blank spaces to note appointment time and preparation plans was also offered as a planning prompt. In addition, households were given the opportunity to request a phone call or in-person reminder prior to their scheduled appointment. This intervention required additional spray staff to accommodate schedules more tailored and responsive to household requests than in previous campaigns.

#### Block leader recruitment (see Table 3)

To address low awareness of neighbors’ participation in the campaign and potential distrust of government campaign staff, this intervention recruited formal and informal community leaders to promote participation and persuade reluctant neighbors. Two to 4 weeks prior to targeted spray dates, campaign staff approached community members who would be known to most people in the neighborhood and asked them to recruit their neighbors to participate in the campaign. These block leaders included both formal community leaders (MOH-trained health promoters, block captains, and elected community officials) and informal neighborhood opinion leaders (corner store owners, daycare coordinators),. Leaders attended training sessions where they were provided with campaign t-shirts, clipboards, mobile airtime cards, and campaign promotional literature. Recruiters were assigned 10–12 households on their block to visit and promote the campaign. This intervention required additional time to recruit, train, and follow-up with leaders, and additional resources to purchase t-shirts, phone cards, and supplies and refreshments for training sessions. The intervention also relied on volunteer time from leaders.

#### Contingent group lotteries (see Table 4)

To address the time and hassle costs of campaign participation, and to encourage communication with neighbors about the campaign, this intervention used contingent group lotteries to reward households for agreeing to participate in the spray campaign. Contiguous households along block faces were assigned to lottery groups of 5–7 households; each group was randomly assigned one lottery number and a specific national lottery drawing date 2–6 weeks in the future. If a group’s number was drawn in the national lottery, households that had participated in the campaign received a voucher for a local hardware store (value PEN 50, $17 at the time of the study). If all of the households in the group had participated, each household received a larger voucher (PEN 100, $34). This intervention required additional staff to promote the lottery during the campaign, and additional resources to purchase and distribute vouchers to winning households.

Feasibility and acceptability of intervention designs were confirmed through rapid-cycle pilots and conversations with Ministry of Health staff. In addition, components of several of the proposed interventions had been tested in prior research undertaken by the team. For example, we had previously piloted narrow-window scheduling and neighbor recruitment during previous campaign phases in four other communities in Arequipa. In this pilot we were able to achieve a participation rate of 92%. Communities had not, however, been randomly assigned to this intervention and were outside the city in an area that may be more open to public health interventions. In terms of prior testing of contingent group lotteries, our team included a leading expert in the use of lotteries for health behavior change. The lottery proposed in our study was adapted directly from a successfully lottery study led by this expert. We also knew from substantial experience in our field site that raffles are commonplace and well-understood in Peru, and are regularly conducted to raise money for schools, cover medical costs, etc.

Over the course of our design phase, there were many interventions that were considered but then rejected for various reasons. The financial incentives arm in particular generated many possible formats and structures for design and delivery of incentives. In the past, district governments have threatened fines for non-participation; to our knowledge, however, these fines have rarely, if ever, been collected. We therefore thought fines would be neither credible nor appropriate. Other suggestions emerging from our analysis that were not within the purview of our project included giving government employees a day off from work to have their houses sprayed, increasing the “opt out” burden of refusal (i.e., requiring households to get a permit to not spray), coordinating with the municipality to give a 10% reduction in local taxes for participating, and changing the insecticide formulation to reduce safety concerns.

### Intervention trial

#### Implementation of designed interventions

In the behavioral design approach, interventions are designed based on identifying actionable bottlenecks and applying behavioral insights to address those bottlenecks. Testing these “idealized” interventions in the field may require adaptations to increase feasibility and acceptability. Unexpected events may also change intervention feasibility. While we had pilot-tested most components of the interventions in earlier studies, our interventions also required real-time modifications in the early phase of the field trial. In Tables 2, 3 and 4 we describe in detail the motivations for and content of the most important and substantive modifications. The pragmatic nature of this trial (i.e. accompanying an existing, ongoing Ministry of Health campaign) required a nimble, responsive approach to intervention implementation, while balancing the critical need for rigor and fidelity in adhering to our study design.

#### Intervention efficacy

The results from this trial are reported in detail elsewhere [41]. Briefly, Cycle 2 participation was high (over 80%) in the study area, with little difference across study arms (see Fig. 1). There was a suggestion of lower participation in the block leader recruitment arm compared with advanced planning and group lotteries in an intent-to-treat sample and lower participation compared with all other arms in a per protocol sample. We assessed two measured of campaign “efficiency”: First, we evaluated whether advanced planning reduced the duration of treatment, given that houses should have been better prepared for treatment. Second, we assessed whether any of the interventions persuaded households to participate after just one campaign visit vs. requiring additional visits during "catch-up" periods. We did not see improved efficiency along either of these dimensions. Finally, a post-hoc assessment of Cycle 2 participation among households who had refused treatment in Cycle 1 revealed significantly higher odds of participation in the advanced planning treatment arm compared with control households.

## Discussion

Our research team is focused on a specific health-related behavior: participation in a government-run insecticide spray campaign to interrupt the vectorial transmission of Chagas disease, a vector-borne parasitic disease. While we were interested in applying principles from behavioral economics to address participation challenges, we were reluctant to show up with a behavioral economics hammer in search of some intervention nails. The behavioral design framework discussed here [12] provided a systematic methodology for defining the behaviors of interest, diagnosing reasons for household reluctance or refusal to participate, designing interventions to address actionable bottlenecks, and then testing those intervention in a rigorous counterfactual context. Behavioral design offered us a broader range of strategies and approaches than pure price changes and community education and health promotion.

Of course, we have to note that the results from our cluster randomized trial of almost 5000 households showed no significant increases in participation for our behaviorally-designed interventions compared to the control campaign [41]. We discuss possible reasons for this finding elsewhere [41], but are reluctant to infer from this one trial that the behavioral design approach is ineffective. The pragmatic, real-world setting of our study made the behavioral design approach challenging, productive, and focused. Behavioral design challenged us to do more—and more different—types of formative work than we had previously conducted in Arequipa around the Chagas campaign. The approach also demanded a new level of collaboration and integration with our partners in the Ministry of Health in order to rethink both the vector control campaign design and implementation processes. We argue that behavioral design was ultimately productive in uncovering previously unstudied explanations for household reluctance and refusal to participate in the campaign, while also keeping the intervention design focused on addressing key actionable bottlenecks rather than letting fashionable or conceptually interesting behavioral insights drive that process. We found that the complexity of an ongoing public health campaign, the interdisciplinary composition of our team, and a long candidate list of compelling and potentially relevant behavioral insights to apply in this setting required a framework that could organize and focus our work in an efficient and transdisciplinary way.

We are not the first team to employ a behavioral design approach in empirical studies. The Datta and Mullainathan framework has been applied to interventions in the agricultural [42], financial services [43–48], WASH (water, sanitation and hygiene) [49, 50] and global health [16, 51] sectors. In addition, numerous governments (although few in developing countries) have instituted “nudge units” or “behavioral insights teams” to bring a behavioral perspective to policy design and implementation [52–54]. It will be important, but challenging, to evaluate the marginal impact of behavioral design on program effectiveness and implementation. One potential evaluation strategy would be random assignment of behavioral design approaches across multiple organizations or sites that are developing and rolling out similar interventions.

An important question going forward is the impact of employing a behavioral design approach on internal and external validity of global health research. In our case behavioral design led to more complex interventions that addressed multiple actionable bottlenecks and employed multiple behavioral insights. Increasingly complex interventions can make interpretation and inference challenging and can make treatment fidelity hard to assess in the field. The implicit pragmatic focus of behavioral design also means that interventions developed through this process are often being deployed in real-world settings where tight control of intervention and sampling is not possible, thereby also undermining internal validity.

Behavioral design can also hinder external validity by tailoring interventions so precisely to the local context through identified actionable bottlenecks that results have limited generalizability to other settings. In our case it is unlikely that the interventions we designed, even if shown to dramatically improve participation in Arequipa’s vector control campaign, would be useful in the same form in another South American Chagas campaign or, for example, in a bed bug elimination campaign in a major US city. This tension is of course not new to the design and evaluation of global health programs and is a key theme in the evaluation revolution. Behavioral design’s contribution to generalizability may therefore lie in the process itself: The define-diagnose-design-test steps can be implemented in virtually any setting for any behaviors of interest, and, if applied rigorously, can expand the use of relevant behavioral insights to solve persistent health challenges.

A related question is how behavioral design approaches may impact scalability of interventions. Scalability refers to the ability to expand successful interventions to a broader population or larger scale [55]. Key dimensions of scalability include cost, workforce to carry out the intervention with fidelity, and interaction of the intervention with contextual factors. Our experience using behavioral design to “supercharge” an existing government-run vector control campaign has convinced us that the approach is a powerful facilitator of scalability, by encouraging strategic tailoring of large-scale interventions to local context. The definition and diagnoses phases of behavioral design can also help to identify the lowest common denominator or minimum effective dose of an intervention prior to scale.

## Conclusions

We applied behavioral design, a four-phase approach to intervention development and evaluation, to the problem of declining household participation in a Chagas disease vector-control campaign in Arequipa, Peru. The approach helped us link problem definition and formative work on diagnosis of actionable bottlenecks to the design and testing of interventions informed by behavioral insights. While we did not follow the prescription to the letter, the framework provided useful guidance. Careful attention to how behavioral design may affect internal and external validity of evaluations, and scalability of interventions, is needed going forward. We recommend behavioral design as a useful complement to other intervention design and evaluation approaches in global health programs.

## Data Availability

The datasets used and/or analysed during the current study are available from the corresponding author on reasonable request.

## Abbreviations

CIDA: Canadian International Development Agency
HIV: Human Immunodeficiency Virus
ITT: Intent-to-treat
MOH: Ministry of Health
PAHO: Pan American Health Organization
RCT: Randomized controlled trial
WHO: World Health Organization
